# Progress on diagnosis and treatment of drug-resistant tuberculosis in line with World Health Organization recommendations in six priority countries in the Western Pacific Region

**DOI:** 10.5365/wpsar.2022.13.4.972

**Published:** 2022-12-19

**Authors:** Kyung Hyun Oh, Maria Imelda Quelapio, Fukushi Morishita, Kalpeshsinh Rahevar, Manami Yanagawa, Tauhid Islam

**Affiliations:** a End TB and Leprosy Unit, World Health Organization Regional Office for the Western Pacific, Manila, Philippines.; b Independent consultant, Cavite, Philippines.

## Abstract

**Background:**

Diagnosis and treatment of drug-resistant tuberculosis (DR-TB) have radically changed in accordance with recommendations from the World Health Organization (WHO) in the past decade, allowing rapid and simple diagnosis and shorter treatment duration with new and repurposed drugs.

**Methods:**

A descriptive analysis of the status and progress of DR-TB diagnosis and treatment in six priority countries in the Western Pacific Region was conducted using information from interviews with countries and the WHO TB database.

**Results:**

Over the past decade, the use of Xpert MTB/RIF has increased in the six priority countries, in parallel with implementation of national policies and algorithms to use Xpert MTB/RIF as an initial diagnostic test for TB and detection of rifampicin resistance. This has resulted in increases in the number of people diagnosed with multidrug-resistant or rifampicin-resistant TB (MDR/RR-TB). Shorter treatment regimens with new and repurposed drugs have also been adopted for MDR/RR-TB cases, alongside a decentralized model of care, leading to improved treatment outcomes.

**Discussion:**

The Western Pacific Region has achieved considerable progress in the diagnosis and treatment of DR-TB, in line with the evolving WHO recommendations in the past decade. The continued commitment of Member States is needed to address remaining challenges, such as the impact of the coronavirus disease pandemic, suboptimal management and health system issues.

Tuberculosis (TB) continues to be a major global health challenge. Despite a concerted global effort to eliminate it, TB remains one of the leading infectious causes of death globally. In 2020, an estimated 10 million incident cases of TB and 1.5 million TB-related deaths occurred worldwide. (*1*) A major threat to the global effort to end TB is drug-resistant TB (DR-TB), which is caused by organisms that are resistant to any drugs used for TB, including multidrug-resistant TB (MDR-TB), which does not respond to isoniazid and rifampicin, the two most effective first-line anti-TB drugs. (*2*) DR-TB is a threat because specialized laboratory infrastructure and diagnostics are required for DR-TB, leading to substantial underdiagnosis of DR-TB, especially in low- and middle-income countries (LMICs); also, treatment outcomes are often poor because treatment of DR-TB is of long duration and involves toxic and expensive medicines. In LMICs, DR-TB can be a huge burden on health systems and can contribute to a high proportion of patients and their families experiencing catastrophic health costs. DR-TB diagnosis and treatment have changed radically in the past decade, in accordance with recommendations from the World Health Organization (WHO), including rapid and simple diagnosis and shorter treatment duration with new and repurposed drugs (**Table 1**). (*3*-*11*)

**Table 1:** WHO recommendations on diagnosis and treatment for DR-TB, 2013–2021

Year	Diagnosis	Treatment
2013	Xpert MTB/RIF to be used as the initial diagnostic test in adults and children suspected of having MDR-TB or HIV-associated TB rather than conventional microscopy, culture and drug susceptibility testing. (*3*)	-
2016	For patients with confirmed RR-TB or MDR-TB, SL-LPA may be used as the initial test to detect resistance to fluoroquinolones, instead of phenotypic culture-based drug susceptibility testing. (*4*)	In patients with RR-TB or MDR-TB who were not previously treated with second-line drugs and in whom resistance to fluoroquinolones and second-line injectable agents was excluded or is considered highly unlikely, a shorter MDR-TB regimen of 9–12 months may be used instead of the longer regimens. (*5*)
2017	Xpert MTB/RIF Ultra is non-inferior to the current Xpert MTB/RIF for the diagnosis of MTB and the detection of rifampicin resistance and can be used as an alternative to the latter in all settings. (*6*)	In patients who require TB retreatment, the category II regimen should no longer be prescribed, and drug susceptibility testing should be conducted to inform the choice of treatment regimen. (*7*)
2018	-	In patients with confirmed rifampicin-susceptible and isoniazid-resistant TB, treatment with rifampicin, ethambutol, pyrazinamide and levofloxacin is recommended for a duration of 6 months. (*8*)
2019	-	In MDR/RR-TB patients on longer regimens, all three Group A agents and at least one Group B agent should be included, to ensure that treatment starts with at least four TB agents likely to be effective and that at least three agents are included for the rest of the treatment after bedaquiline is stopped. If only one or two Group A agents are used, both Group B agents are to be included. If the regimen cannot be composed with agents from Groups A and B alone, Group C agents are to be added. (*9*)
	-	Kanamycin and capreomycin are not to be included in the treatment of MDR/RR-TB patients on longer regimens. (*9*)
2020	-	A shorter all-oral bedaquiline-containing regimen of 9–12 months duration is recommended in eligible patients with confirmed MDR/RR-TB who have not been exposed to treatment with second-line TB medicines used in this regimen for more than 1 month, and in whom resistance to fluoroquinolones has been excluded. (*10*)
	-	A treatment regimen lasting 6–9 months, composed of bedaquiline, pretomanid and linezolid (BPaL), may be used under operational research conditions in MDR-TB patients with TB that is resistant to fluoroquinolones, who have either had no previous exposure to bedaquiline and linezolid or have been exposed for no more than 2 weeks. (*10*)
2021	In people with bacteriologically confirmed pulmonary TB, low complexity automated NAATs may be used on sputum for the initial detection of resistance to isoniazid and fluoroquinolones, rather than culture-based phenotypic drug susceptibility testing. (*11*)	-

MDR-TB: multidrug-resistant tuberculosis; NAAT: nucleic acid amplification test; RR-TB: rifampicin-resistant tuberculosis; SL-LPA: second-line line probe assay; TB: tuberculosis; Xpert MTB/RIF: an automated NAAT for *Mycobacterium tuberculosis* complex and resistance to the drug rifampicin.

Group A: levofloxacin/moxifloxacin, bedaquiline and linezolid.

Group B: clofazimine and cycloserine/terizidone.

Group C: ethambutol, delamanid, pyrazimide, imipenem-cilastatin, meropenem, amikacin/streptomycin, ethionamide/prothionamide and *p*-aminosalicylic acid.

The WHO Western Pacific Region is home to 1.9 billion people in 37 countries and areas. The region is diverse, ranging from a large country with a population of more than 1 billion to small Pacific island countries with a few thousand residents, and from countries with a high TB burden to countries in the pre-elimination stage. (*12*) Five countries (China, Mongolia, Papua New Guinea, the Philippines and Viet Nam) in the region are on the WHO global list of 30 countries with a high burden of TB and multidrug-resistant or rifampicin-resistant TB (MDR/RR-TB) for 2021–2025. (*13*) Countries and areas of the region have striven to adopt the changing WHO recommendations for the diagnosis and treatment of DR-TB. The regional Green Light Committee (rGLC), established in 2011 as a regional DR-TB advisory committee to WHO, has supported the scale-up of the programmatic management of DR-TB (PMDT) in countries with a high MDR/RR-TB burden in the region. (*14*) This analysis provides an overview of the status and progress of DR-TB diagnosis and treatment in six priority countries in the Western Pacific Region in line with WHO recommendations.

## Methods

The Western Pacific regional framework to end TB: 2021–2030 indicates 10 priority countries in the region. (*15*) Six countries that are directly supported by rGLC – Cambodia, the Lao People's Democratic Republic, Mongolia, Papua New Guinea, the Philippines and Viet Nam – were selected for this descriptive analysis of the status and progress of DR-TB diagnosis and treatment using information from interviews with countries and the WHO TB database.

Indicators to assess current diagnosis and treatment processes for DR-TB were based on recommendations in the latest WHO guidelines; they included:

use of Xpert MTB/RIF, Xpert MTB/RIF Ultra (Xpert Ultra) and Xpert MTB/XDR for diagnostic tests;
use of shorter all-oral bedaquiline-containing regimens;
discontinuation of kanamycin and capreomycin for MDR/RR-TB; and
use of a bedaquiline, pretomanid and linezolid (BPaL) regimen. (*10*, *11*)

Indicators to assess the progress of diagnosis and treatment for DR-TB were from the latest WHO global TB reports; they included:

percentage of TB patients tested for rifampicin resistance;
proportion of people diagnosed with MDR/RR-TB and enrolled in MDR-TB treatment in the same year (this can be more than 100% owing to cases that are enrolled in the year after they are diagnosed);
percentage of MDR/RR-TB cases tested for susceptibility to fluoroquinolones;
number of MDR/RR-TB cases treated with bedaquiline, shorter regimen and all-oral longer regimen; and
treatment outcomes for MDR/RR-TB cases started on treatment. (*1*, *16*)

Information on the countries’ status was obtained through interviews, supported by follow-up communication and rGLC country monitoring missions. Between July and November 2021, interviews with PMDT focal points of the national TB programmes from the six priority countries were conducted virtually using a structured questionnaire. Further information was updated after follow-up communications or subsequent rGLC country monitoring missions in 2021. Information on the countries’ progress was collected from the WHO database, to which countries and areas annually report data on TB care and prevention via an electronic platform. (*17*)

All data analyses and visualizations were conducted using the statistical software package R 4.1.1 (Comprehensive R Archive Network at https://cran.r-project.org/).

## Results

### Status of DR-TB diagnosis

The six priority countries are at different stages in their uptake of WHO recommendations on diagnostic tools and algorithms for DR-TB (**Table 2**). National policies and algorithms indicate universal access to drug susceptibility testing in all priority countries. Although Xpert MTB/RIF is used as an initial diagnostic test for TB and rifampicin resistance detection in all priority countries, in Cambodia and Viet Nam it does not cover the entire population. In Cambodia, Xpert MTB/RIF was used for all initial diagnostic testing in some areas; in other areas, it was limited to high-risk groups, such as previously treated cases and people living with HIV. The country aims to expand its universal use to all presumptive cases nationwide by 2023. In Viet Nam, Xpert MTB/RIF is limited to high-risk groups such as people living with HIV, children and people with abnormal lesions on chest X-ray. In priority countries, the number of GeneXpert machines per 1 million population ranges from three to 12.7 nationwide.

**Table 2:** Status of DR-TB diagnosis in priority countries in the Western Pacific Region in 2021

Item	Cambodia	Lao People's Democratic Republic PDR	Mongolia	Papua New Guinea	Philippines	Viet Nam
Xpert MTB/RIF used as an initial diagnostic test for TB and rifampicin resistance detection	In some areas (all areas by 2023)	Yes	Yes	Yes	Yes	Yes (only for high-risk groups and people with abnormal lesion on chest X-ray)
National policy and algorithm indicate universal access to drug susceptibility testing	Yes	Yes	Yes	Yes	Yes	Yes
Number of GeneXpert machines (per 1 million population)	107 (6.4)	57 (7.8)	42 (12.7)	85 (9.6)	587 (5.4)	285 (3.0)
Use of Xpert Ultra	Replacing Xpert MTB/RIF since 2019	Together with Xpert MTB/RIF since 2021	Together with Xpert MTB/RIF since 2021	Together with Xpert MTB/RIF since 2017	Together with Xpert MTB/RIF since 2020	Together with Xpert MTB/RIF since 2018
Use of Xpert MTB/XDR	No	Planned in 2022	No	Planned in 2022	Planned in 2022	Planned in 2022
FL-LPA used to detect isoniazid resistance among rifampicin-susceptible TB	Yes (ad hoc since 2019)	No	No	No	No	No
FL-LPA used to detect isoniazid resistance among rifampicin-resistant TB	Yes (ad hoc since 2018)	No	Yes (routinely since 2016)	Yes (routinely)	Yes (routinely since 2021)	Yes (ad hoc since 2016)
SL-LPA used as an initial test to detect fluoroquinolone resistance among confirmed MDR/RR-TB	Yes (since 2017)	Yes (since 2016)	Yes (since 2016)	Yes	Yes (since 2017)	Yes (since 2016)
Use of Truenat	Planned as a pilot in 2022	No	No	No	Planned as a pilot in 2022	Planned as a pilot in 2022
Phenotypic drug susceptibility testing for new and repurposed drugs	No	Lzd (Bdq, Cfz, Dlm planned)	Bdq, Cfz, Lzd	No	No	Bdq, Cfz, Dlm, Lzd (Pa planned)

Bdq: bedaquiline; Cfz: clofazimine; Dlm: delamanid; FL-LPA: first-line line probe assay; Lzd: linezolid; MDR/RR-TB: multidrug-resistant tuberculosis or rifampicin-resistant tuberculosis; Pa: pretomanid; PDR: People’s Democratic Republic; SL-LPA: second-line line probe assay; TB: tuberculosis; Xpert MTB/RIF: an automated nucleic acid amplification test for  *Mycobacterium tuberculosis* complex and resistance to the drug rifampicin; Xpert Ultra: Xpert MTB/RIF Ultra.

Xpert MTB/RIF Ultra (Xpert Ultra) has been introduced in all priority countries. In Cambodia, Xpert Ultra had already replaced Xpert MTB/RIF at the time of the interview. In the other priority countries, Xpert Ultra is being used together with Xpert MTB/RIF. There are also plans to introduce Xpert MTB/XDR in 2022 in the Lao People's Democratic Republic, Papua New Guinea, the Philippines and Viet Nam.

In Cambodia, first-line line probe assays (FL-LPAs) are used to detect isoniazid resistance among rifampicin-susceptible TB cases only on an ad hoc basis. However, in Mongolia, Papua New Guinea and the Philippines, FL-LPAs are routinely used to detect isoniazid resistance among rifampicin-resistant TB cases, and in Cambodia and Viet Nam, they are used in such cases but on an ad hoc basis. Second-line line probe assays (SL-LPAs) are used as an initial test to detect fluoroquinolone resistance among confirmed MDR/RR-TB cases in all priority countries. The use of Truenat (a point-of-care rapid molecular test) for detection of TB and rifampicin resistance is planned as a pilot project in 2022 in Cambodia, the Philippines and  Viet Nam.

As the use of new and repurposed drugs in shorter and longer regimens is scaled up in priority countries, phenotypic drug susceptibility testing for those drugs is conducted or planned. In the Lao People's Democratic Republic, drug susceptibility testing for linezolid is in place, and drug susceptibility testing for bedaquiline, clofazimine and delamanid is planned. In Mongolia, drug susceptibility testing for bedaquiline, linezolid and clofazimine is conducted. In Viet Nam, drug susceptibility testing for bedaquiline, linezolid, clofazimine and delamanid is conducted and drug susceptibility testing for pretomanid is planned.

### Progress of DR-TB diagnosis

**Fig. 1:**
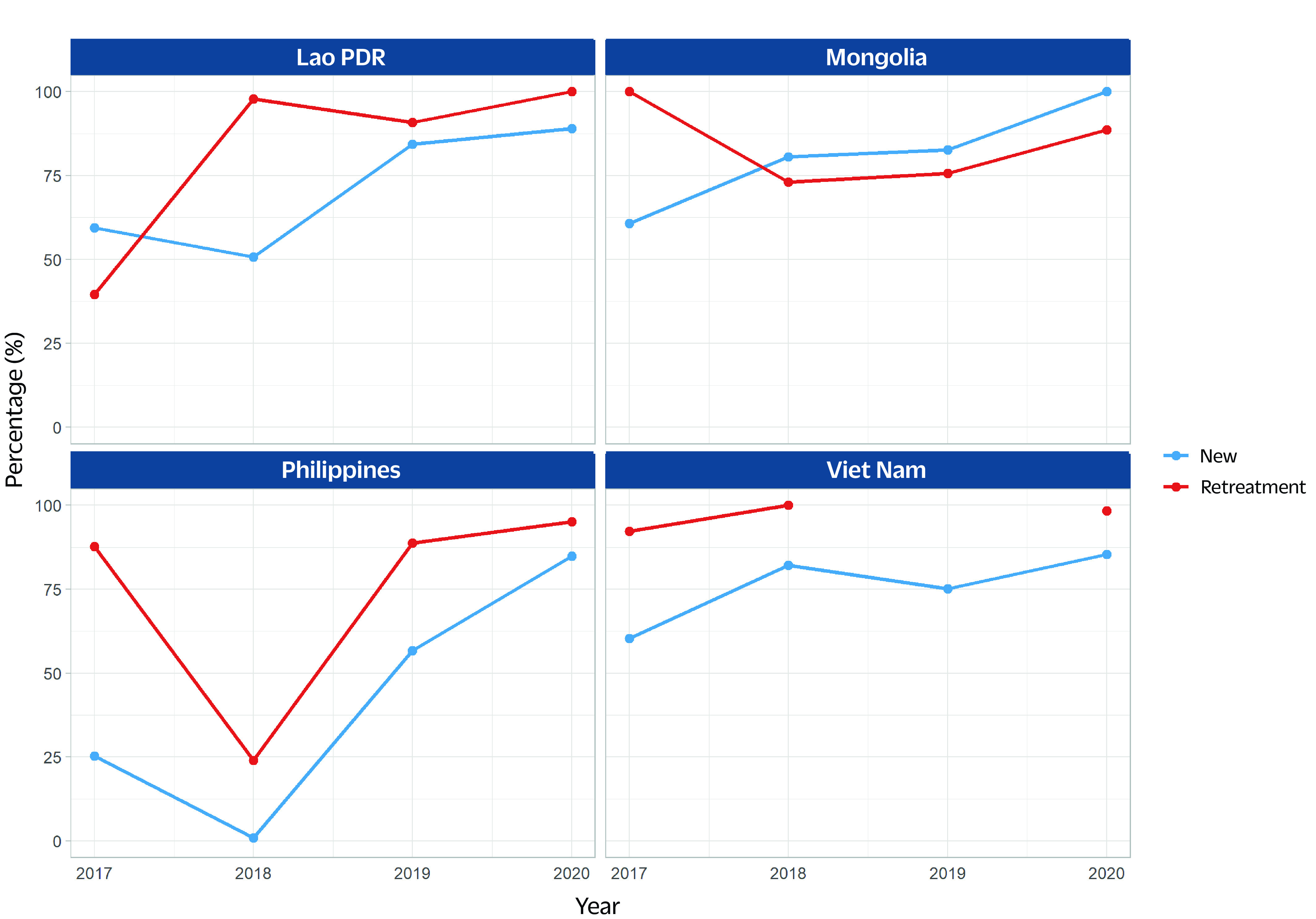
Percentage of new and previously treated TB patients tested for rifampicin resistance in four priority countries, 2017–2020 (no reports from Cambodia and Papua New Guinea)

**Fig. 2:**
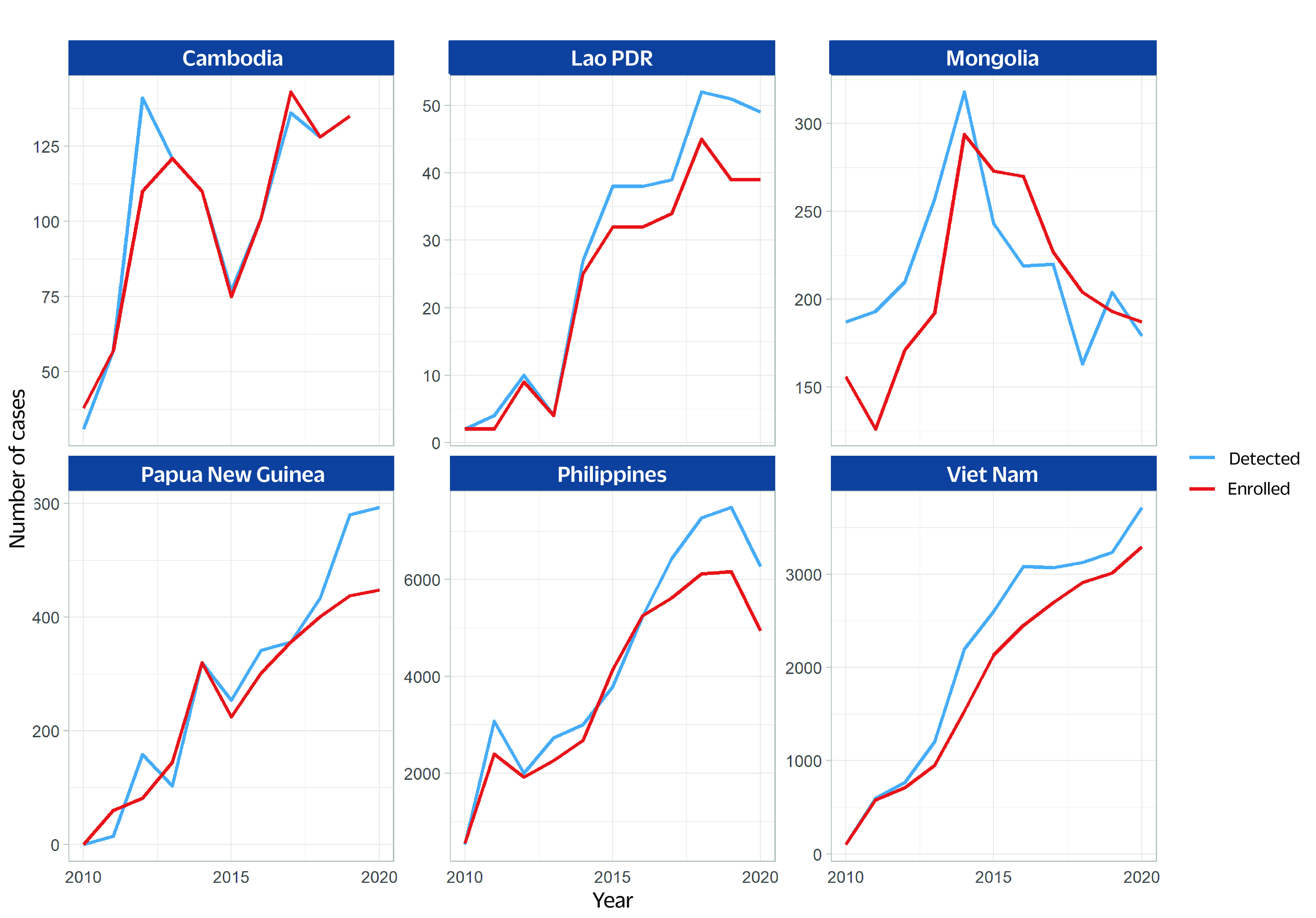
Number of people diagnosed with MDR/RR-TB and enrolled in MDR-TB treatment in six priority countries, 2010–2020

The percentage of new and previously treated TB patients tested for rifampicin resistance in four of the priority countries (no reports from Cambodia and Papua New Guinea) increased between 2017 and 2020, with some fluctuations (**Fig. 1**). The number of people diagnosed with MDR/RR-TB and the number starting on treatment per year in all countries has increased since 2010  (**Fig. 2**). However, in the Lao People's Democratic Republic, Mongolia and the Philippines, there was a decrease in the number of MDR/RR-TB cases diagnosed in 2020 compared with 2019. This decrease started in 2014 in Mongolia. The proportion of enrolment in treatment among diagnosed cases exceeded 80% in 2020 in Mongolia (104%) and Viet Nam (89%), whereas it was 80% or below in the Lao People's Democratic Republic (80%), Papua New Guinea (76%) and the Philippines (78%).

[**insert** Figure 1]

[**insert** Figure 2]

**Fig. 3:**
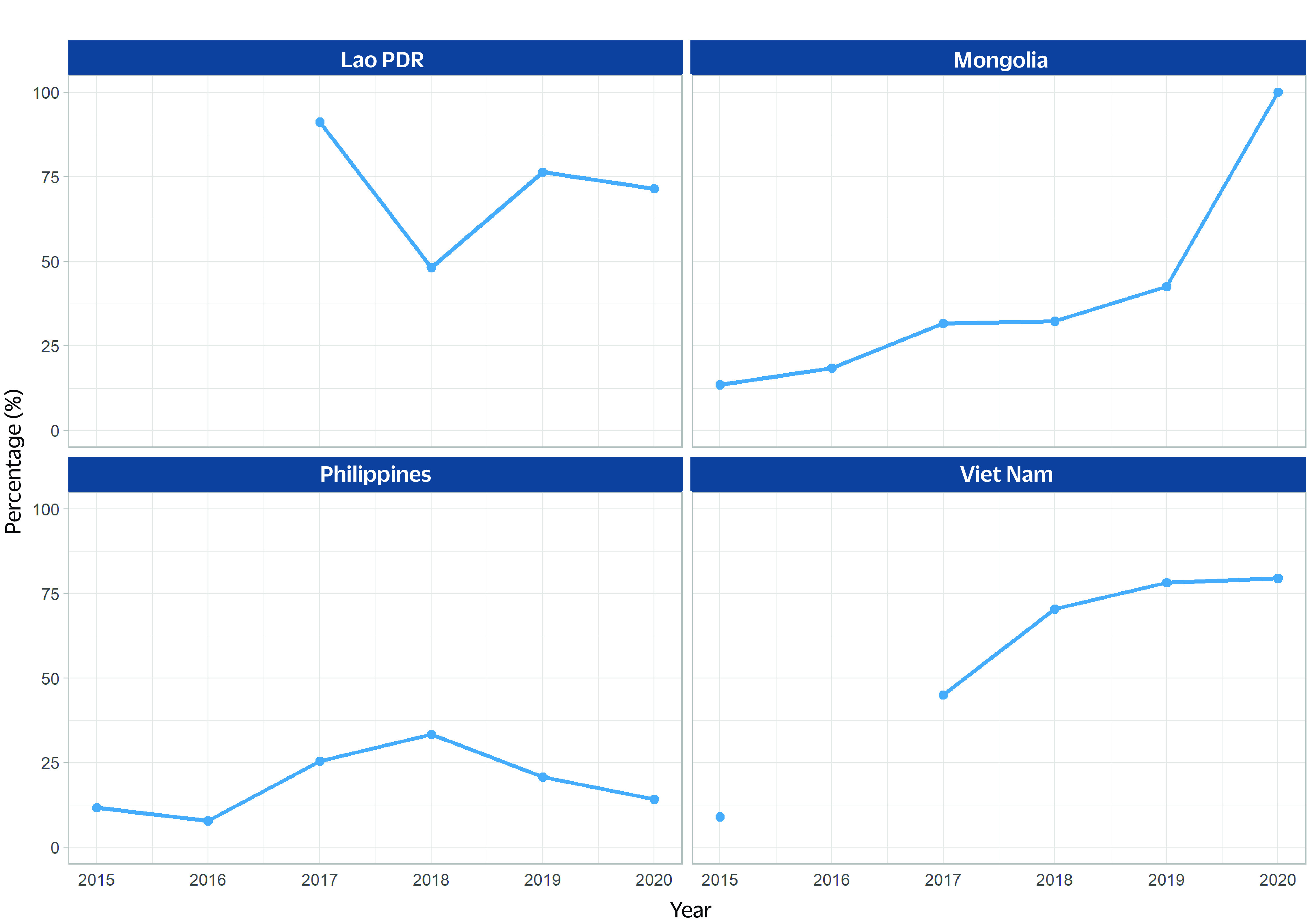
Percentage of MDR/RR-TB cases tested for susceptibility to fluoroquinolones in four priority countries, 2015–2020 (no reports from Cambodia and Papua New Guinea)

The percentage of MDR/RR-TB cases tested for susceptibility to fluoroquinolones in four priority countries between 2015 and 2020 (no reports from Cambodia and Papua New Guinea) varied with increased coverage in Mongolia and Viet Nam (**Fig. 3**).

[**insert** Figure 3]

### Status of DR-TB treatment

The six priority countries are also at different stages in the uptake of WHO recommendations for DR-TB treatment (**Table 3**). The category II regimen for retreatment cases has been discontinued in all priority countries. However, the WHO-recommended regimen for rifampicin-susceptible and isoniazid-resistant TB (Hr-TB) is used only in Cambodia, Mongolia, Papua New Guinea and Viet Nam.

**Table 3:** Status of DR-TB treatment in priority countries in the Western Pacific Region in 2021

Item	Cambodia	Lao People's Democratic Republic PDR	Mongolia	Papua New Guinea	Philippines	Viet Nam
Discontinuation of category II regimen	Yes (in 2020)	Yes (in 2018)	Yes (in 2020)	Yes (in 2017)	Yes (in 2017)	Yes (in 2018)
6 REZ-Lfx for Hr-TB	Yes (since 2018)	No	Yes (since 2020)	Yes (since 2020)	No	Yes (since 2018)
Shorter injectable-containing regimen	2017–2021	2013–2020	2016–2020	Yes (since 2016)	2015–2021	2016–2020
Shorter all-oral bedaquiline-containing regimen	Yes (since 2020)	Yes (since 2020)	Yes (since 2020)	No	Yes (since 2020)	Yes (since 2021)
Standardized longer regimen for fluoroquinolone-susceptible MDR/RR-TB	6 months Bdq-Lfx-Lzd-Cfz/12–14 months Lfx-Lzd-Cfz or 12–14 months Lfx-Cfz-Cs	N/A (shorter all-oral Bdq-containing regimen solely used)	6 months of 2–3 in Group A & 1–2 in Group B & 1–2 in Group C/ 12–14 months of 1–2 in Group A & 1–2 in Group B & 1–2 in Group C	6 months Bdq-Lfx-Lzd-Cfz/12 months Lfx-Lzd-Cfz	6 months Lfx-Bdq-Lzd-Cfz/12–14 months Lfx-Lzd-Cfz	Bdq-Lfx-Lzd-Cfz-1 in Group C or Lfx-Lzd-Cfz-Cs-1 in Group C
Standardized longer regimen for fluoroquinolone-resistant MDR/RR-TB	6 months Bdq-Lzd-Cfz-Cs-Dlm/12–14 months Lzd-Cfz-Cs	N/A (BPaL solely used)		N/A (individualized)	6 months Bdq-Lzd-Cfz-Cs/12–14 months Lzd-Cfz-Cs	N/A (individualized)
Discontinuation of kanamycin and capreomycin for MDR/RR-TB	Yes (in 2020)	Yes (in 2019)	Yes (in 2020)	Yes (in 2020)	Yes (in 2019)	Yes (in 2020)
Unavailable drugs in longer regimens	Ipm-Cln, Mpm	Cs, Ipm-Cln, Mpm, S, PAS	None	Ipm-Cln, Mpm	Mpm	Mpm
Use of BPaL regimen	No	Yes (under OR conditions since 2020)	Yes (under programmatic conditions since 2021)	No	Yes (under OR conditions since 2021)	Yes (under OR conditions since 2021)
Culture monitoring for MDR/RR-TB	Monthly	Monthly	Monthly	Irregular due to laboratory instability	Monthly	Monthly
Elective partial lung resection	No	No	Yes	No	Yes	No
Treatment adherence interventions	Material support (US$ 30 per month) Psychological support Patient education Staff education	Material support (US$ 5 per day) Psychological support Patient education Staff education	Material support (lunch & transportation) Psychological support Patient education Staff education	Material support (US$ 57–100 per month) Psychological support Patient education Staff education	Material support (US$ 18 per week) Patient education Staff education Digital medication monitor (pilot)	Material support (US$ 10 per month) Patient education Staff education Digital medication monitor (pilot)
Treatment administration options	Community-based DOT by health-care workers or family members	Facility-based DOT by health-care workers	Facility-based DOT by health-care workers Community-based DOT by family members VOT (pilot)	Facility-based DOT by health-care workers Community-based DOT by family members	Facility-based DOT by health-care workers Community-based DOT by family members VOT (pilot)	Community-based DOT by health-care workers or family members VOT (pilot)
Model of care	Mainly ambulatory care	Mainly hospitalization	Hospitalization until sputum conversion followed by ambulatory care	Mainly ambulatory care	Mainly ambulatory care	Hospitalization up to 1 month followed by ambulatory care

Bdq: bedaquiline; BPaL: bedaquiline, pretomanid and linezolid; Cfz: clofazimine; Cs: cycloserine; Dlm: delamanid; DOT: directly observed treatment; E: ethambutol; Hr-TB: rifampicin-susceptible, isoniazid-resistant tuberculosis; Ipm-Cln: imipenem-cilastatin; Lfx: levofloxacin; Lzd: linezolid; MDR/RR-TB: multidrug-resistant tuberculosis or rifampicin-resistant tuberculosis; Mpm: meropenem; OR: operational research; PAS: p-aminosalicylic acid; PDR: People’s Democratic Republic; R: rifampicin; REZ-Lfx: rifampicin, ethambutol, pyrazinamide and levofloxacin; S: streptomycin; VOT: video-observed treatment; Z: pyrazinamide.

Group A: levofloxacin/moxifloxacin, bedaquiline and linezolid.

Group B: clofazimine and cycloserine/terizidone.

Group C: ethambutol, delamanid, pyrazimide, imipenem-cilastatin, meropenem, amikacin/streptomycin, ethionamide/prothionamide and p-aminosalicylic acid.

Shorter injectable-containing regimens started in all priority countries between 2013 and 2017. In accordance with the 2020 update of the WHO guidelines on DR-TB treatment, (*10*) the shorter all-oral bedaquiline-containing regimen has been used in Cambodia, the Lao People's Democratic Republic, Mongolia, the Philippines and Viet Nam since 2020, with the shorter injectable-containing regimen being phased out accordingly.

As per the 2019 WHO consolidated guidelines on DR-TB treatment, (*9*) standardized longer regimens for fluoroquinolone-susceptible or fluoroquinolone-resistant MDR/RR-TB have been revised in Cambodia, Mongolia, Papua New Guinea, the Philippines and Viet Nam, prioritizing Group A drugs including bedaquiline and linezolid. Kanamycin and capreomycin were no longer included in the treatment of MDR/RR-TB patients on longer regimens in all priority countries by 2020. Among the medicines recommended for use in longer regimens, (*9*) the following drugs are unavailable: imipenem-cilastatin and meropenem in Cambodia; cycloserine, imipenem-cilastatin, meropenem, streptomycin and p-aminosalicylic acid in the Lao People's Democratic Republic; none in Mongolia; imipenem-cilastatin and meropenem in Papua New Guinea; meropenem in the Philippines; and meropenem in Viet Nam.

The BPaL regimen recommended in 2019 for operational research has been used in the Lao People's Democratic Republic, the Philippines and Viet Nam since 2020 or 2021. Mongolia commenced the BPaL regimen under programmatic conditions in 2021.

Culture monitoring for MDR/RR-TB patients on treatment is conducted monthly in all priority countries except for Papua New Guinea. Elective partial lung resection (lobectomy or wedge resection) alongside a recommended MDR-TB regimen is being undertaken in Mongolia and the Philippines.

Various treatment adherence interventions for MDR/RR-TB patients are offered in the six priority countries (**Table 2**). Material support (e.g. lunch, transport and cash transfer) is provided in all priority countries. Psychological support is offered in Cambodia, the Lao People's Democratic Republic, Mongolia and Papua New Guinea. Patient and staff education is provided in all priority countries. Digital medication monitoring has been implemented as a pilot project in the Philippines and Viet Nam.

Several treatment administration options for MDR/RR-TB patients are provided in priority countries. In Cambodia and Viet Nam, the main mode of treatment administration is community-based directly observed treatment (DOT) by health-care workers or family members. In Mongolia, Papua New Guinea and the Philippines, both facility-based DOT by health-care workers and community-based DOT by family members are used. In the Lao People's Democratic Republic, the main modality is facility-based DOT by health-care workers during hospitalization. Video-observed treatment (VOT) has been conducted as a pilot project in Mongolia, the Philippines and Viet Nam.

MDR/RR-TB patients are treated mainly through ambulatory care in Papua New Guinea and the Philippines, whereas most patients are treated during hospitalization in the Lao People's Democratic Republic. In Mongolia, patients are hospitalized until their sputum conversion from positive to negative, and in Viet Nam, patients are hospitalized for up to 1 month. In Cambodia, patients were hospitalized for the first week for a workup and monitoring of a new regimen before the coronavirus disease (COVID-19) pandemic; however, this practice has since been restricted to ambulatory care only.

### Progress of DR-TB treatment

**Fig. 4:**
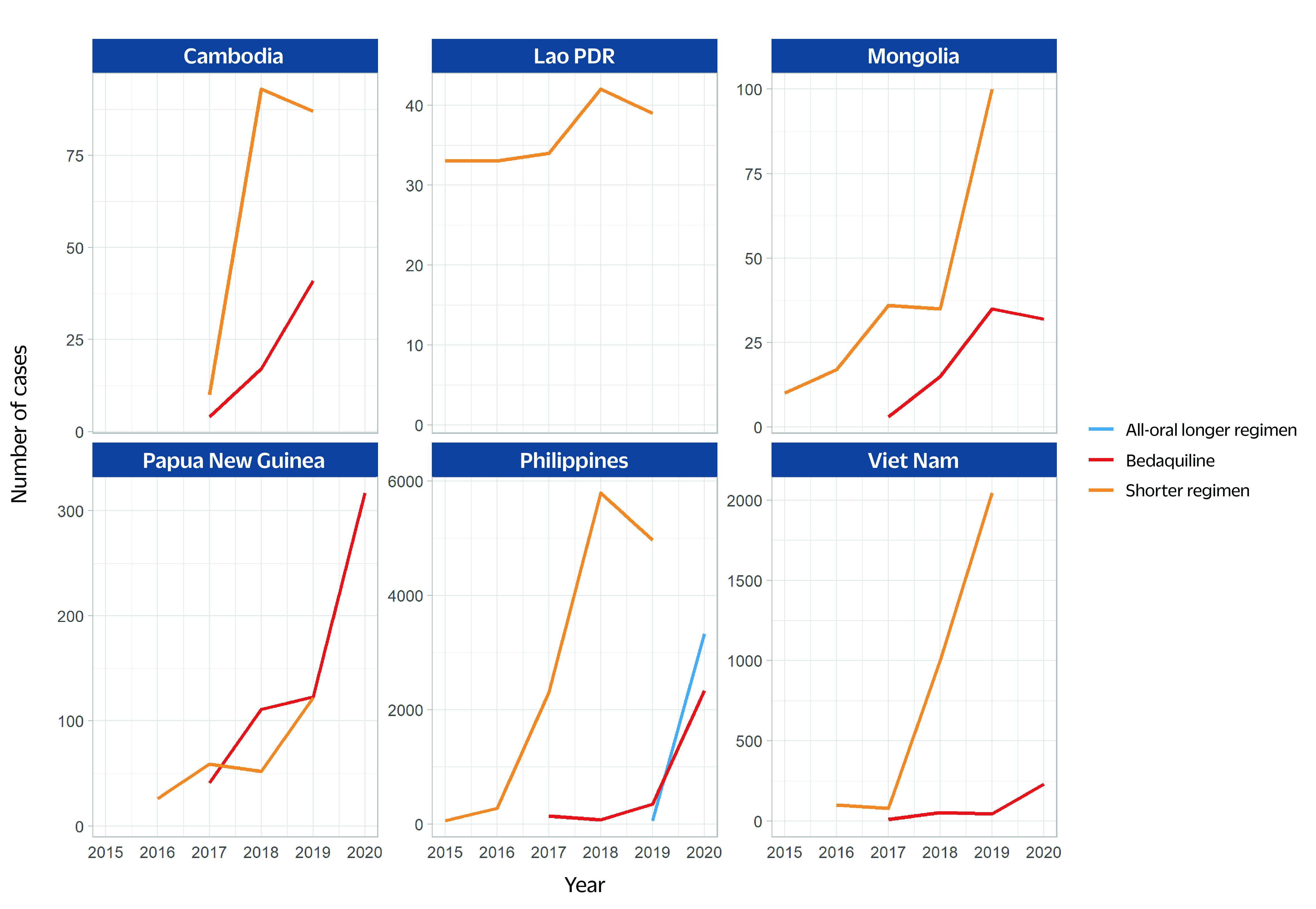
Number of MDR/RR-TB cases treated with all-oral longer regimen, bedaquiline and shorter regimen in six priority countries, 2015–2020

Where data were available, the number of MDR/RR-TB cases treated with bedaquiline and the shorter regimen between 2015 and 2019 increased in all six priority countries, although there were decreases observed between 2018 and 2019 in Cambodia, the Lao People's Democratic Republic and the Philippines (**Fig. 4**). The use of the all-oral longer regimen increased between 2019 and 2020 in the Philippines (no report from the other countries).

[**insert** Figure 4]

**Fig. 5:**
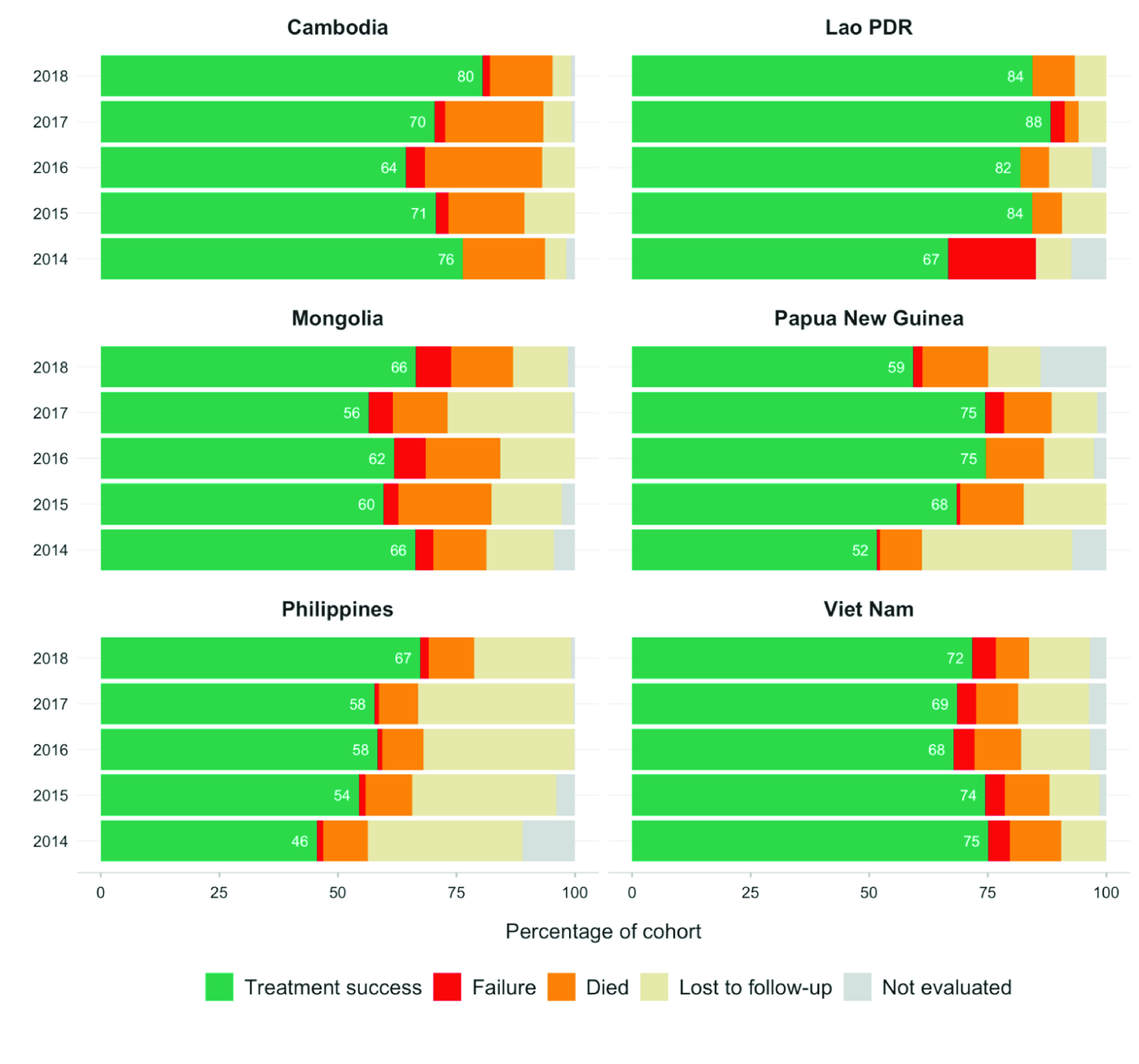
Treatment outcomes for MDR/RR-TB cases started on treatment in six priority countries, 2014–2018

Treatment outcomes for MDR/RR-TB cases started on treatment in 2014–2018 in the six priority countries differed (**Fig. 5**). The proportion of cases with treatment success increased in the Lao People's Democratic Republic (from 67% in 2014 to 84% in 2018) and the Philippines (from 46% in 2014 to 67% in 2018), mainly due to a reduction in the proportion of treatment failure in the Lao People's Democratic Republic and to patient loss to follow-up in the Philippines. The proportion of cases with treatment success was similar each year in Mongolia and Viet Nam, and fluctuated from year to year in Cambodia and Papua New Guinea.

[**insert** Figure 5]

## Discussion

DR-TB diagnosis and treatment in the Western Pacific Region have changed drastically over the past decade. As shown by the six priority countries, national policies and algorithms now recommend Xpert MTB/RIF as an initial diagnostic test for TB and rifampicin resistance detection; also, the number of Xpert MTB/RIF sites and GeneXpert machines has increased, with the eligibility criteria extended to all people to be evaluated for TB. As a result, the number of people diagnosed with MDR/RR-TB and the percentage of TB patients tested for rifampicin resistance have also increased. For DR-TB treatment, shorter regimens for MDR/RR-TB treatment are used, with the shorter all-oral bedaquiline-containing regimen replacing the shorter injectable-containing regimen. New and repurposed drugs have been included in shorter or longer regimens, and kanamycin and capreomycin have been withdrawn. The model of care for MDR/RR-TB treatment is transitioning towards decentralization and increased use of ambulatory care. Consequently, the treatment outcomes for MDR/RR-TB cases have improved in these priority countries.

The rGLC has supported the scale-up of programmatic management of DR-TB in priority countries. (*14*) In accordance with WHO recommendations, the committee has provided technical inputs to national strategies or guidelines related to DR-TB; assisted in national capacity-building activities (e.g. in-person workshops or webinars); and conducted annual rGLC monitoring missions, where members of the committee and WHO staff have provided recommendations and have monitored countries’ actions to previous recommendations. (*14*, *18*) Therefore, most of the new diagnostics and regimens have been implemented in a timely manner in the priority countries.

COVID-19 has impacted the diagnosis and treatment of TB and DR-TB since 2020. (*19*) The decrease in the number of MDR/RR-TB cases diagnosed in the Lao People's Democratic Republic, Mongolia and the Philippines in 2020 can be attributed to the decrease in TB notifications due to restricted visits to health facilities and the repurposing of TB staff and facilities for the COVID-19 response. GeneXpert machines have been repurposed for COVID-19 diagnosis; also, stockouts of cartridges and a lack of equipment maintenance may have contributed to the decrease in MDR/RR-TB diagnoses. Despite these difficulties, COVID-19 has facilitated innovations in DR-TB treatment. (*20*) All priority countries have expedited the transition from injectable-containing shorter or longer regimens to all-oral shorter or longer regimens, to minimize patient visits to health facilities. Hospitalization for treatment and facility-based DOT has been minimized, and community-based DOT has been facilitated in all priority countries. Also, the use of digital technology such as VOT was accelerated in three priority countries during the pandemic.

This analysis shows that there is still a gap in enrolment in treatment for cases diagnosed with MDR/RR-TB in some priority countries. Programme staff in the field have reported that this might be due to several factors, including death or loss to follow-up of cases before treatment commencement; significant delay in diagnosis due to the long turnaround time of SL-LPA or phenotypic drug susceptibility testing results; and delay in treatment due to health system challenges including drug supply interruption, stockouts or insufficient staff capacities, or inaccuracies in recording and reporting from paper-based systems. There should be a focus on addressing these factors for more timely patient treatment enrolment in those countries.

Although SL-LPA has been successfully promoted as initial drug susceptibility testing for fluoroquinolones in all priority countries, for various reasons, coverage is suboptimal in some of these countries. One issue is that there may be an insufficient number of laboratories for SL-LPA for nationwide coverage. For example, in the Philippines, a large country, there was only one laboratory functional for SL-LPA between 2020 and 2021 from among three designated laboratories, because one facility was repurposed for COVID-19 testing and another had never started SL-LPA testing. It has been reported from the field that SL-LPA may be underused in areas where Xpert MTB/RIF-confirmed RR-TB patients are initiated on MDR-TB treatment without SL-LPA testing against national guidelines. There may also be missing data on SL-LPA results during the recording and reporting process. With the Lao People's Democratic Republic, Papua New Guinea, the Philippines and Viet Nam soon to introduce Xpert MTB/XDR, a new landscape of drug susceptibility testing for fluoroquinolones is expected.

Due to the underuse of FL-LPA and regimens for Hr-TB in most priority countries, diagnosis and treatment of Hr-TB remain limited in the region. FL-LPA or the soon-to-be-introduced Xpert MTB/XDR should be further used to detect isoniazid resistance in cases of rifampicin-susceptible TB (at least among retreatment cases), given the high rate of isoniazid resistance in some countries. Moreover, the regimen for Hr-TB should be implemented in more of the priority countries.

There was static and fluctuating MDR/RR-TB treatment success in some priority countries, despite the roll-out of shorter regimens. Although they are shorter, these regimens do not guarantee improved treatment outcomes because they still require clinical management, strong patient support and monitoring systems to ensure patient adherence to treatment. In those countries, expansion of shorter regimens should be reinforced by optimal management and supportive health systems for improved treatment outcomes.

Our analysis has several limitations. First, there were no reported data for some indicators for certain years or from particular countries in the WHO database, impeding a complete analysis. Second, there is a possibility of recall bias from PMDT focal points, despite verification by follow-up communication or subsequent rGLC missions. Although attempts were made to refer to official documents, some answers were provided based on memory. Notwithstanding these limitations, this analysis provides a comprehensive and practical insight into the progress of PMDT in these six priority countries in the region.

In conclusion, these six priority countries in the Western Pacific Region, in collaboration with the rGLC, have achieved considerable progress in the diagnosis and treatment of DR-TB in line with the evolving WHO recommendations over the past decade. Automated nucleic acid amplification tests and shorter all-oral regimens containing new and repurposed drugs are now used for DR-TB diagnosis and treatment in the region, leading to reductions in the case-detection gap and enhanced treatment outcomes. However, several challenges remain, particularly the impact of the COVID-19 pandemic, suboptimal patient management and health system issues. The continued commitment of countries to a speedy recovery from COVID-19, patient-centred care, capacity building and a robust health system is needed to continue progressing towards ending DR-TB in the region.
